# Analyzing force measurements of multi-cellular clusters comprising indeterminate geometries

**DOI:** 10.1007/s10237-023-01764-9

**Published:** 2023-09-28

**Authors:** Yifat Brill-Karniely, Katerina Tischenko, Ofra Benny

**Affiliations:** 1 Institute for Drug Research, The School of Pharmacy, Faculty of Medicine, The Hebrew University of Jerusalem, 9112001 Jerusalem, Israel; 2 Institute of Animal Science, ARO, The Volcani Center, 50250 Bet-Dagan, Israel

**Keywords:** Biomechanics, Tumor spheroids, Force spectroscopy, Mathematical regression, Data analysis, Young’s modulus

## Abstract

Multi-cellular biomimetic models often comprise heterogenic geometries. Therefore, quantification of their mechanical properties—which is crucial for various biomedical applications—is a challenge. Due to its simplicity, linear fitting is traditionally used in analyzing force—displacement data of parallel compression measurements of multi-cellular clusters, such as tumor spheroids. However, the linear assumption would be artificial when the contact geometry is not planar. We propose here the integrated elasticity (IE) regression, which is based on extrapolation of established elastic theories for well-defined geometries, and is free, extremely simple to apply, and optimal for analyzing coarsely concave multi-cellular clusters. We studied here the quality of the data analysis in force measurements of tumor spheroids comprising different types of melanoma cells, using either the IE or the traditional linear regressions. The IE regression maintained excellent precision also when the contact geometry deviated from planarity (as shown by our image analysis). While the quality of the linear fittings was relatively satisfying, these predicted smaller elastic moduli as compared to the IE regression. This was in accordance with previous studies, in which the elastic moduli predicted by linear fits were smaller compared to those obtained by well-established methods. This suggests that linear regressions underestimate the elastic constants of bio-samples even in cases where the fitting precision seems satisfying, and highlights the need in alternative methods as the IE scheme. For comparison between different types of spheroids we further recommend to increase the soundness by regarding relative moduli, using universal reference samples.

## Introduction

Reliable comparison between the mechanical properties of three-dimensional (3D) bio-samples is needed in many biotechnological applications, such as tissue engineering, diagnostics and drug design (Brill-Karniely et al. 2020; Dixon et al. 2018; Chaudhuri et al. 2020). Prime parameters of interest are the elastic moduli, being intrinsic measurable properties which describe the elastic response of the samples to small mechanical perturbations. The elastic moduli are obtained by fitting stress-strain measurements to physical models (Brill-Karniely 2020; Kilpatrick et al. 2015). In such experiments force is exerted while recording the sample deformation. It is often desirable to avoid local sampling, and for that aim platen indenters that are larger than the sample are useful, allowing for parallel compression of the whole object. This way, collective information can be gained, accounting for example for both cells and ECM components. A major factor which determines the stress–strain relation is the change in the contact area between the sample and the probe during the experiment. Therefore, the physical theories are very sensitive to the rest geometry of the sample.

Tumor spheroids are 3D aggregates composed of cancer cells with external or internal ECM components, and in some cases they contain additional cells such as stromal cells, providing a useful ex vivo model of tumors with a micro-environmental context (Shoval et al. 2017; Weiswald et al. 2015). Due to the tight link between the mechanical properties of the cells and the cancerous potential, the mechanics of tumor spheroids is of significant relevance to fields of cancer diagnostics and rational drug design (Brill-Karniely et al. 2020; Suresh 2007; Boot et al. 2021). Tumor spheroids (and other multi-cellular clusters), are often macroscopically concave, and comprise geometrical variations between them even when composed of the same number and type of cells (Brill-Karniely et al. 2020; Shoval et al. 2017). To date there is no accessible data fitting scheme which captures different spheroid geometries. Such scheme would be crucial both for analyzing experimental repetitions of the same spheroid kind, and also for reliable comparison between varying types of spheroids.

**Fig. 1:**
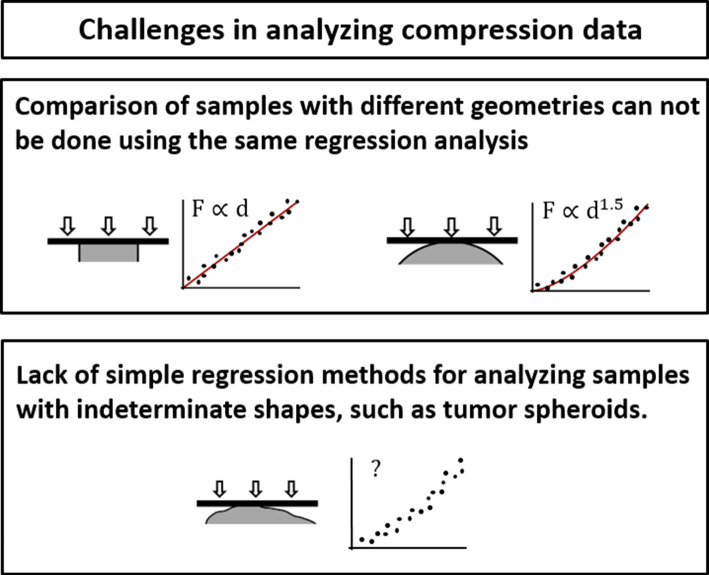
Challenges in the analysis of force spectroscopy data of parallel compression. Top: Simple analytical analysis, such as linear regression (left) or Hertz model (right) capture only the elasticities of specific well-defined shapes. Thus samples that do not share the same geometry cannot be analyzed using the same model. Bottom: There is lack in user friendly regression methods that can be easily applied for the analysis of elastic bodies whose shape is not well defined, such as multi-cellular clusters

If constant parallel contact was maintained during the compression experiment, the displacement would be linearly proportional to the force. However, such ideal conditions are not relevant for cellular clusters, largely due to their complex geometry. In spite of that, linear force—displacement fitting is widely used in the data analysis of cellular clusters (Conrad et al. 2019; Omelyanenko et al. 2020; Pradhan et al. 2017; Baraniak et al. 2012). Physical models which do account for variations in the sample—probe contact area are provided by theories from the field of contact mechanics (Kilpatrick et al. 2015; Popov 2013). For example, the Hertz model can be used for fitting parallel compression data of spherical samples (Jiménez-Piqué et al. 2014). Different variations of contact mechanics theories regard specific well-defined geometries of the bodies in contact. Thus when using contact mechanics for fitting force data of samples that do not share the same (well defined) shape, various models may be needed, as illustrated in Fig. 1 (top). This raises a conflict when aiming to compare between the mechanical properties of the samples, even if they share the same composition. One option would be to use different models for each sample; however this reduces the reliability of the comparison. The second option is to use the same model assumptions for analyzing all measurements, yet this may be artificial for some of the samples. The difficulties are pronounced not only when aiming to perform sample comparison but also when studying the mechanical properties of a sample whose shape poorly suits the geometrical assumptions of existing analytical theories (Fig. 1, bottom). In this case, fitting the data according to models that require specific shape assumptions may be doubtable.

In the present work, in order to overcome these challenges, we constructed the integrated elasticity (IE) scheme. The IE regression is based on extrapolating analytical three-dimensional (3D) theories into a higher dimensional model. The IE model is ideal for analyzing force spectroscopy data of multi-cellular clusters or other 3D bodies with no topological irregularities as long as their surface is approximately concave, and the macroscopic curvature of their upper margin, which is in contact with the probe, is between planar to spherical (Fig. 1, bottom). A large advantage of the IE method is that it provides a single regression that is suitable for data analysis of non-identical spheroids (or other samples) that are macroscopically concave.

Traditionally, the physical models used in elastic measurements of 3D bodies are frictionless contact mechanics models, often being the default analysis methods in the built-in software of force spectroscopy instruments such as Atomic Force Microscopy (AFM) (Brill-Karniely 2020; Kilpatrick et al. 2015). Using linear or contact mechanics theories, the elastic moduli are obtained by fitting data from sample squeezing (when the probe is approaching rather than retracting the sample), where viscosity effects can be negligible (Efremov et al. 2017). While versions of frictionless contact mechanics/linear theories differ in their specific assumptions, all of these models, whether directly or under some minor approximations, can be expressed as power laws (Brill-Karniely 2020):

1 $$F\left(\delta \right)=EC{\delta }^{\alpha }$$

where *E* represents the elastic constant, *F* is the force and $\delta$ the displacement. The coefficient *C* and the exponent $\alpha$ vary between the different models and depend on the system geometry. Other theoretical methods such as finite element theory, various computer simulations or complex numerical and analytical models are infrequently used as they require unique expertise and larger efforts, or that they are not accessible for free (Hajji 1978; Vahabikashi et al. 2019; Karcher et al. 2003).

As noted above, an ideal case is that of a constant planar and parallel contact with no adhesive interactions. Then Eq. 1 can be written with $C=\frac{A}{h}$ and $\alpha =1$ where *A* is the contact area and *h* the sample thickness in equilibrium. Due to its simplicity, this linear regression is often used for analysing three-dimensional (3D) mechanical data using a planar indenter, even for samples whose shape is not well defined, such as tumor spheroids (Conrad et al. 2019; Omelyanenko et al. 2020; Pradhan et al. 2017; Baraniak et al. 2012). Linear regression in force measurements of tumor spheroids was previously performed by fitting either all force—displacement data (within small deformations), or only as little as two data points that are chosen at the onset of the force increase (Conrad et al. 2019; Omelyanenko et al. 2020; Pradhan et al. 2017; Baraniak et al. 2012). While the validity of both methods is questionable, the latter case is poorer since the fit largely depends on the choice of the two data points.

**Fig. 2:**
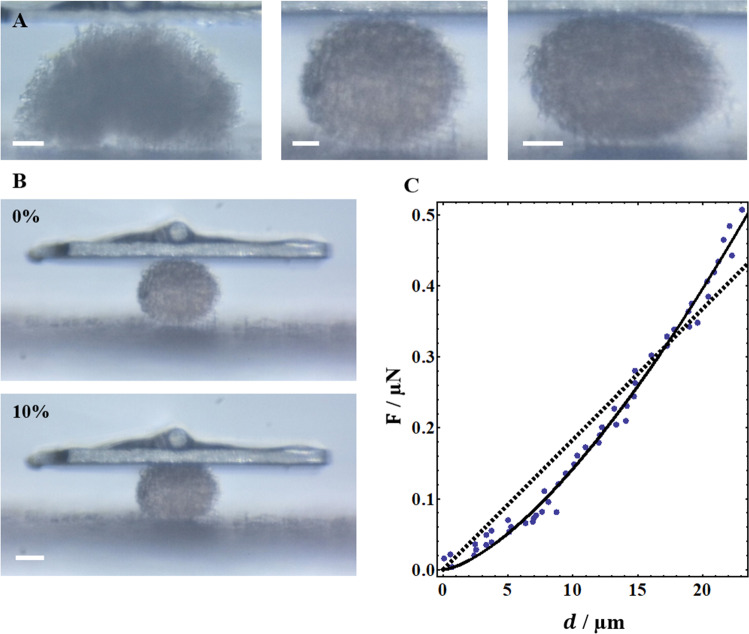
**A** Tumor spheroids vary in their geometry. Shown are spheroids of A375 (left, bar = 100 μm) and WM-266–4 (middle and right, bar = 50 μm). **B** Force—displacement data of 10% squeezing was analyzed using the linear (dashed) or IE (solid) models (**C**). Panels B and C show results obtained in a WM-266-4 spheroid, for which the value of α in the IE fit was 1.47. Bar in panel *B* = 100 μm

In this work we investigated the quality of data analysis in force measurements of tumor spheroids constructed from two different cell lines of human melanoma: A375 and WM-266-4. It is well known that tumor and cell biomechanics strongly correlates with the cancer aggressiveness (Brill-Karniely et al. 2020; Suresh 2007; Wirtz et al. 2011). Comparing the mechanical properties of different tumor spheroids is thus very relevant for biomedical applications (Brill-Karniely et al. 2020). However, the shape of the spheroids is non-uniform, even within the same cell type, as demonstrated in Fig. 2A. It is thus important to clarify what is the reliability of different analyzes methods in this case.

Our image analysis revealed that in both regressions higher precision was obtained for spheroids comprising smooth contour and planar margin. In those cases, variations in the contact area during the measurement would be minimal. In spheroids of relatively smooth surfaces, the precision maintained high values using the IE fit (unlike the linear model), even when there was large deviation from planarity. In general, we showed here that the IE analysis provided better accuracy than the linear one when fitting force—displacement data of tumor spheroids. While *R*^2^ values of the linear fit were also satisfying, it predicted *E* values that were more than twofold smaller than those obtained by the IE regression. This raised a general conflict about the reliability of experimental data analysis: high accuracy of data fitting does not necessarily indicate on the validity of the parameters obtained by the analysis. For reliable information we suggest to regard relative values using a universal reference, such as polyacrylamide gels that were used here.

## Materials and methods

### Tumor spheroid formation

Spheroids were prepared as detailed in our previous work (Steinberg et al. 2020). Master 3D Petri dish 35-well arrays (Microtissues Inc., Providence, RI, USA) were used to create micro-wells made of 2% UltraPureTM agarose hydrogel (Invitrogen, Carlsbad, CA, USA). Micro-wells were seeded with 5,000 A375 or WM-266-4 cells per well. A375 and WM-266-4 cells were purchased from ATCC. The cells were incubated at 37 °C and 5% CO_2_ for 72 h allowing for the growth of tumor spheroids.

### MicroTester measurements of tumor spheroids

Mechanical properties of A375 and WM-266-4 spheroids were measured in a parallel-plate compression configuration using the CellScale MicroTester LT with a stainless steel plate. The spheroids were compressed by 10% of their rest height (see Fig. 2B). The duration of each compression was 30 s. A 0.1524 mm beam was used with a modulus of 411,000 MPa. The width and height of the spheroids at rest were obtained using the MicroTester software. The data analysis (either linear or IE) was carried out using Wolfram Mathematica software.

### The integrated elasticity (IE) regression

The IE regression aimed to obtain a sole expression for fitting data of elastic force measurements in multi-cellular (or other) bodies of non-uniform geometries, whose macroscopic curvature is between planar and spherical. It is based on a mathematical extrapolation which uses the solutions of flat and spherical contacts as boundary conditions and assumes a smooth analytical transition between these two cases. The samples are assumed to be concave on macroscopic scale, with a curvature that is roughly between planar and spherical along their contact with the planar indenter, and without topological irregularities. The common assumptions of contact mechanics are relevant also here, namely that the process is elastic and that the samples are homogeneous and isotropic on macro-scale. Moreover, adhesive and friction interactions are neglected due to the inert nature of the surfaces in contact with the spheroids. The elastic modulus *E* is defined by $\frac{1}{E}=\frac{1-{\upsilon }^{2}}{\widetilde{E}}$ where $\widetilde{E}$ and $\upsilon$ are, respectively, the Young’s modulus and Poisson’s ratio of the sample, and it is assumed that the indenting plate is infinitely rigid.

In an ideal case where the upper sample contour is planar and parallel to the plate, the stress–strain relation theory obeys Eq. 1 with $C=\frac{A}{h}$ and $\alpha =1$ (Popov 2013). Then, assuming a cylindrical sample of diameter $x$ one obtains:

2 $$F=\frac{\pi {x}^{2}}{4h}E\bullet \delta $$

On the other hand, when the sample is spherical the Hertz model yields Eq. 1 to be valid with $C=\frac{4}{3}{R}^{0.5}$ (where *R* is the sphere radius) and $\alpha =1.5$:

3 $$F=\frac{4}{3}E{R}^{0.5}{\delta }^{1.5}$$

Equations 2 and 3 provide the boundary conditions of the IE regression, where Eq. 1 is used as a general form. For the pre-exponential factor a simple power law expression was assumed, providing a smooth transition between the planar and spherical solutions for $\alpha =$ 1 or 1.5, respectively:

4 $$C\left(\alpha ,x, h\right)=2\left[(1.5-\propto ){\left(\frac{\pi }{4}\right)}^{\left(3-2\alpha \right)}+(\propto -1){\left(\frac{4}{3\sqrt{2}}\right)}^{(2\alpha -2)}\right]\bullet {\left(\frac{{x}^{2}}{h}\right)}^{2-\alpha }$$

In this expression $x$ and $h$ are, respectively, the width and height of the sample (for a sphere both $x$ and $h$ equal 2*R*), where it is assumed that $x$ represents both the width and length of the sample. $\alpha$ is obtained from the fit of each sample independently, and reflects properties of the object geometry in a generalized manner. This allows fitting the data without pre-assumption of the exponent, which may not be uniformly appropriate for all samples. The reference experiments were done with a cubic gel. In this case $x$ and $h$ were equal to the effective diameter of a sphere comprising the same volume as the cube. The linear fit in the case of the cubic gel was done using $F=LE\delta$, where *L* was the cube length. Additional details about elastic measurements of the gels were recently published (Tischenko et al. 2023).

### Geometrical analysis

The rest geometry of the upper 10% of each tested spheroid was analysed using MATLAB. The deviation from macroscopic planarity was defined as $\chi \equiv \sqrt{\frac{1}{N}{\sum }_{\left\{i\right\}}{\left({y}_{i}-<y>\right)}^{2}}/\left(h<y>\right)$, whereas for each spheroid the normalized ‘non-planarity’ was given by the ratio ${\chi }_{\mathrm{spheroid}}/{\chi }_{\mathrm{circle}}$. In this expression *y*_*i*_ was the *y* coordinate of the *i*_th_ index in the array of true pixels, where the *y* axis was defined as parallel to the direction of the force. *N* was the number of pixels in this array and *h* the height of the sample. Furthermore, for a more microscopic geometrical insight, we calculated the smoothness of the spheroids. Also here the upper 10% of spheroid surface was analyzed. We defined the smoothness as $l/d$ where $l$ was the end to end distance of the contour in *x* direction and *d* the length of the border line.

## Results

**Fig. 3:**
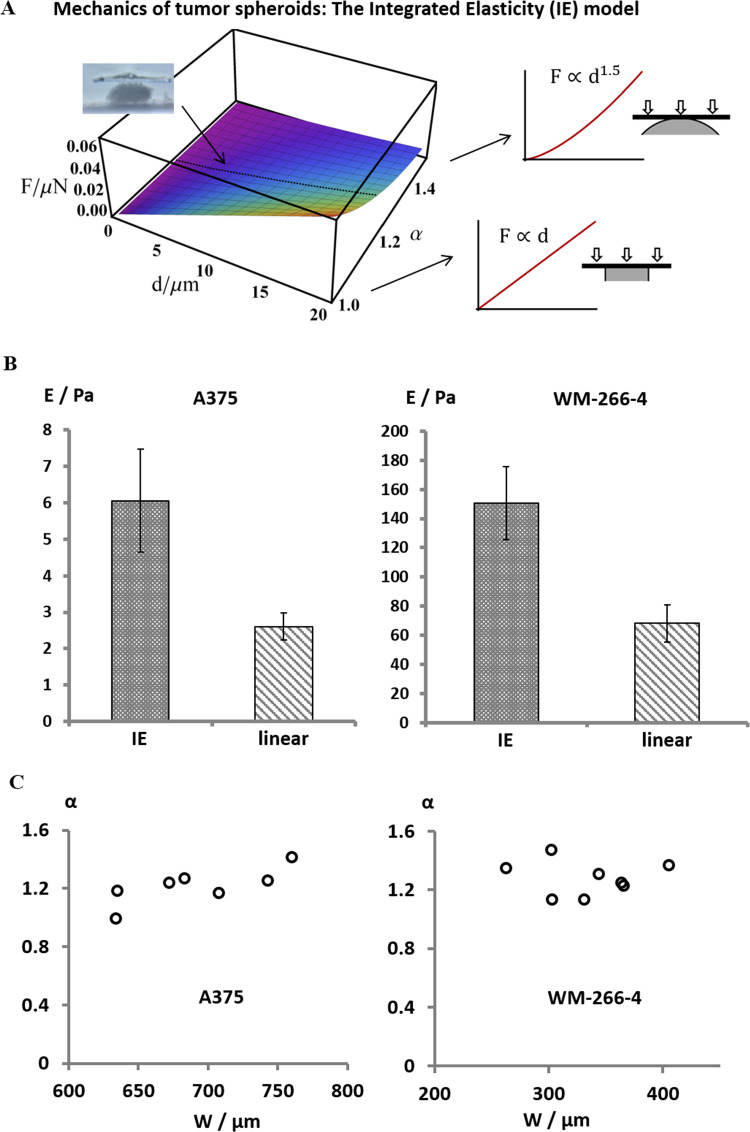
**A** The Integrated Elasticity (IE) theory can describe the mechanics of 3D bodies whose shape is not well defined, and requires only coarse geometrical assumptions. The exponent *α* is achieved for each individual sample. The boundary conditions of the model are planar and spherical contacts. **B** The elastic moduli of both A375 and WM-266-4 tumor spheroids were predicted to be more than twofold higher using the IE regression relative to the linear fit. **C** As predicted, α did not depend on the spheroid width

Force measurements using MicroTester (Cell Scale LTD) were performed in two types of spheroids composing different types of human melanoma cell lines: either A375 or WM-266-4. In order to safely remain under elastic conditions, we analyzed data of 10% spheroid squeezing (Fig. 2B), after which the spheroids returned to their original geometry following a gradual release of the force. Figure 2C demonstrates that even within this limited regime the linear fit (dashed curve) was not optimal. The solid fit in Fig. 2C, which provided better accuracy, is based on the integrated elasticity (IE) regression. The boundary conditions of the IE model are provided by pre-solved theories of linear and spherical contacts, as explained in the Methods section and presented in Fig. 3A. As illustrated in the figure, for each spheroid the exponent α was independently achieved and the elastic modulus was derived based on Eq. 1 and Eq. 4. As expected, the obtained values of α for the spheroids tested were from 1 to 1.5, namely between the solutions of planar and spherical contacts.

Data analysis of the two types of tumor spheroids using either the linear or the IE regressions revealed different values of spheroid elastic moduli. Figure 3B shows that both for A375 and for WM-266-4 the linear model predicted spheroid moduli that were more than twofold smaller than those obtained by the IE theory. This is in accordance with inconsistency that was previously found in the comparison between linear regressions and well established analysis methods, as further elaborated here in the Discussion (Baraniak et al. 2012; Conrad et al. 2019; Tietze et al. 2019). Using both analyses we found here that A375 spheroids were one order of magnitude more elastic than the WM-266-4 spheroids. This signifies that spheroids composing different cell types can vary a lot from each other in factors which dictate their mechanical properties, such as the spheroid density, the presence of ECM components, the 3D organization of cells, the cell–cell and cell-ECM contacts etc. Figure 3C shows that the values of $\alpha$ are not related to the thickness of the spheroids. This was predicted as the physics of the system does not depend on the sample size, as long as the spheroids comprise a macroscopic number of cells, on the one hand, and are smaller than the indenting plate on the other hand.

**Fig. 4:**
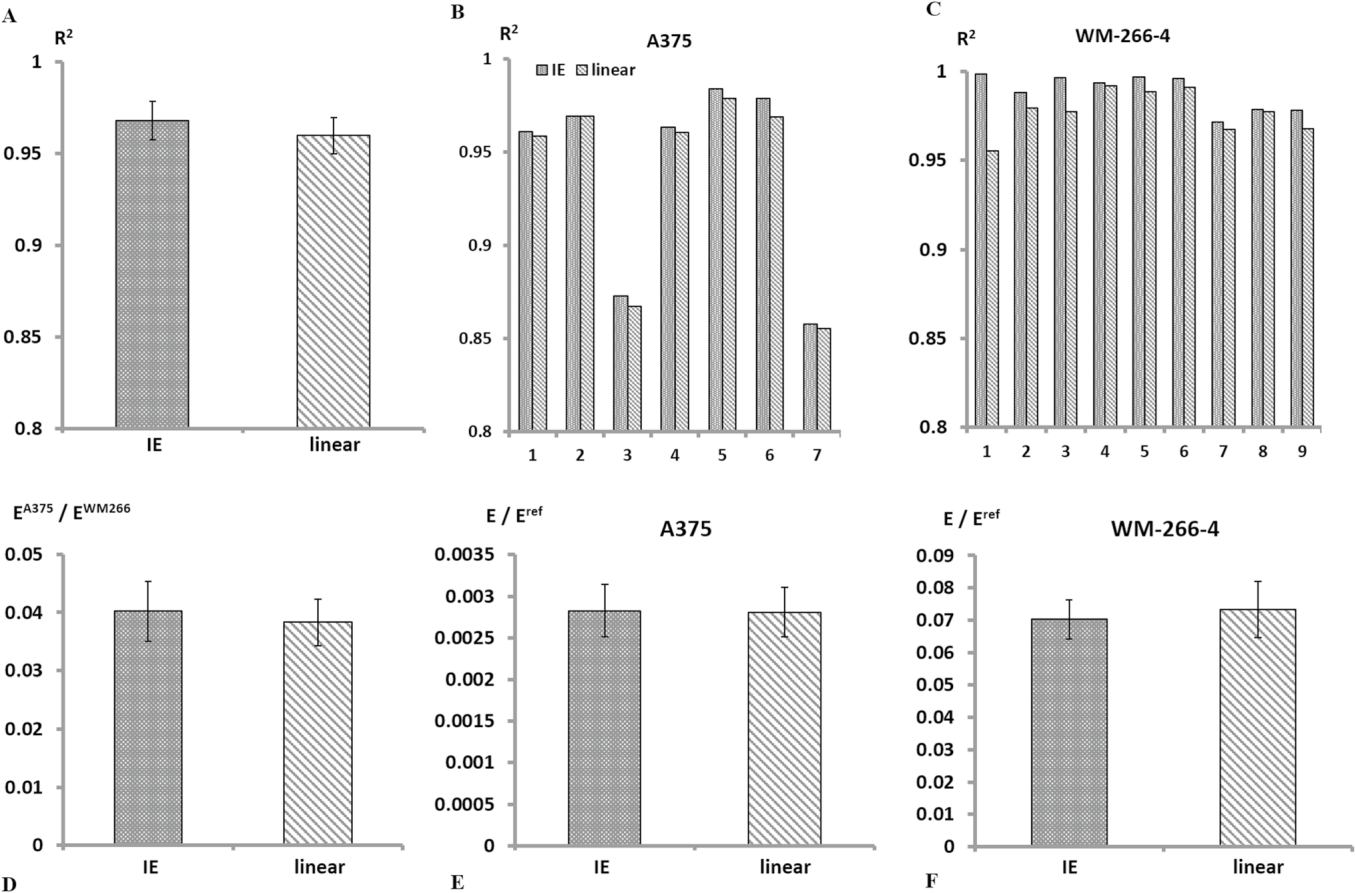
**A** Averages of *R*^2^ values obtained in the analysis of spheroid force- displacement data using either the linear or the IE regressions. Individual precisions for A375 or WM-266-4 spheroids are shown in panels (**B**) and (**C**) respectively. **D** The relative elasticity of the two spheroid types did not depend on the choice of the model. **E–F** Universal values can be obtained using a polyacrylamide gel as a standard reference. Using this reference, the relative E estimates for both A375 (**E**) or WM-266-4 (**F**) spheroids did not depend on the type of the model

The lower values predicted by the linear model as compared to other methods, both in our work and in previous studies, raise the need in examining the accuracy of the data fitting (Baraniak et al. 2012; Conrad et al. 2019; Tietze et al. 2019). Figure 4A–C show that as predicted the IE model provided higher values of *R*^2^, however the accuracy of the linear fit was also of satisfactory. This observation highlights an important conflict: high precision of data fits does not necessarily indicate on the validity of the analysis: different models which provide fits of high quality can predict diverse parameter evaluations.

What should be done then for a reliable evaluation of elastic constants from parallel compression of 3D samples? This question is challenging since validation needs to be determined by comparison to well-established measurements; however it is not trivial to determine which working schemes, including analysis methods, can provide valid reference values. In the case of tumor spheroids, theories that are considered to be well established were used for analyzing various types of experiments (Boot et al. 2021; Tietze et al. 2019; Dague et al. 2017). However, to the best of our knowledge, these did not include spheroid compression by parallel plates, in which case linear regression was used (Omelyanenko et al. 2020; Pradhan et al. 2017; Baraniak et al. 2012; Conrad et al. 2019). Due to the lack of established reference we suggest here to regard relative (rather than absolute) values of the Young’s moduli, when aiming to compare between different types of spheroids using parallel compression experiments. One option of a relative value that we examined here was the ratio between the spheroid elastic constants of the two cell types, *E*^*A375*^/*E*^*WM266*^, where each was analyzed using the same regression. Interestingly, Fig. 4D shows that these ratios did not depend on the type of the model. Namely, the relative values were consistent with each other, unlike the absolute moduli.

While the ratio between elastic moduli of different sample types can sometimes be of interest, there is an important need also in achieving universal information that is not specific to a given experiment. For that aim we suggest to use well-defined reference samples. Then, one can calculate the ratio *E*/*E*^ref^ between the elastic moduli of the tested sample and that of the reference sample, after both were analyzed using the same model. Here we used polyacrylamide gels as standard references, that were fabricated following an established protocol of 1 kPa rigidity (Fischer et al. 2012). We tested gel cubes with the MicroTester and analyzed the data using both the IE and the linear regressions. The relative values obtained using this method were found not to depend on the model type, as shown in panels *E* and *F* of Fig. 4, for A375 or WM-266-4 spheroids respectively. This strengthened the notion that the soundness of the comparison between the elasticities of varying sample types can be increased when using a universal reference, such as the polyacrylamide gels used here. Moreover it raises the option that the linear approximation may be applicable for obtaining relative elastic moduli, rather than absolute ones.

**Fig. 5:**
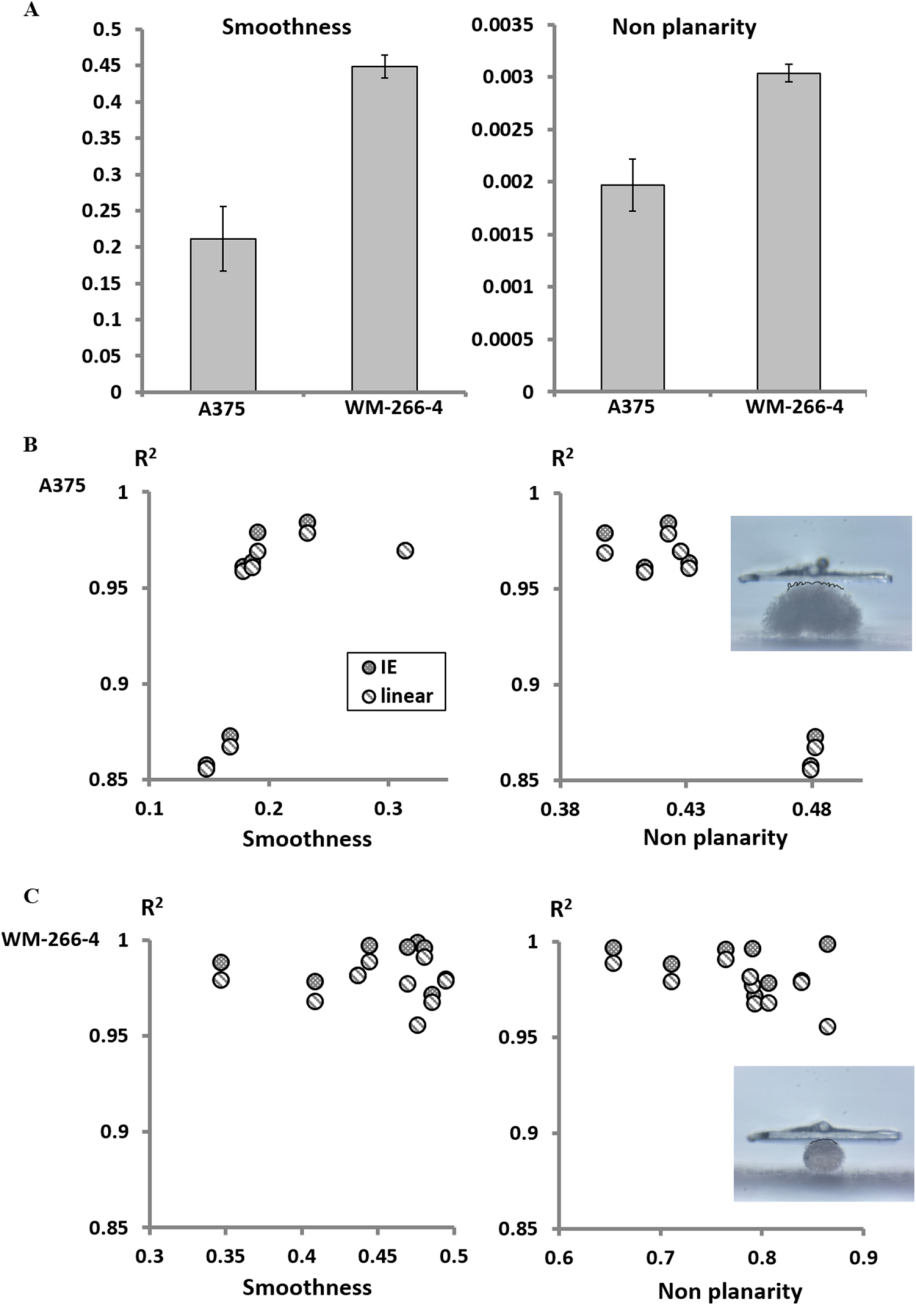
**A** Image analysis revealed that the A375 spheroids were rougher than WM-266-4 ones and that their upper surface was more planar. *R*^2^ values of both the IE and the linear fittings are plotted as a function of the smoothness and the non-planarity for spheroids composed of either A375 cells (**B**) or WM-266-4 cells (**C**)

To investigate the dependence of the model precision on the shape of the sample, we performed image analysis of the spheroids using MATLAB. Since the linear model assumes constant smooth and planar contact, we calculated the smoothness and the planarity of the upper 10% contour of the spheroids. We found that the WM-266-4 spheroids were smoother than the A375 ones, hinting that they may have higher suitability to the linear regression (Fig. 5A, left). Conversely, on a larger length scale outlook, the upper surfaces of the WM-266-4 spheroids was less planar than those of A375, suggesting they may be less compatible to the linear fit (Fig. 5A, right).

Aiming to reveal the relation between the fit precision to the spheroid shape we plotted the R^2^ values of single spheroids as a function of the spheroid smoothness and the non-planarity, for A375 (Fig. 5B) and WM-266 (Fig. 5C). For A375 spheroids it was clear that the lower *R*^2^ values were correlated both with the spheroid roughness and with the deviation from macroscopic planarity. Two of the A375 spheroids tested had apparent lower *R*^2^ values. Figure 5B shows that those spheroids were both rougher and had less planar surfaces than the others. The right plot in Fig. 5C shows that in WM-266-4 spheroids, which have relatively smooth surfaces, the reduction in *R*^2^ with the non-planarity was minor in the case of the IE model, relative to the linear fit. This highlights the potential strength of the IE model relative to the linear one for smooth objects which do not have a planar contour.

## Discussion

With the accumulating knowledge related to the role of cell and tissue mechanics in normal function or in pathological processes, there is a growing need in gaining reliable and comparative biomechanical information. However, the complexity of biological samples places a challenge in performing simple universal analysis of biomechanical data. This difficulty is mostly pronounced when aiming for large scale information, which can provide collective mechanical knowledge about the whole sample, such as in the MicroTester experiments done here. Then the indenter is larger than the sample and the contact area is affected by the complex morphology of the sample boundary. In contrast, in AFM measurements the indenter dimensions are much smaller than cell size. Thus, the information gained is local, and describes specific nano-regimes within the spheroids, depending on the pressing location and on the depth of the indentation (Vyas et al. 2019; Gnanachandran et al. 2022; Kosheleva et al. 2023; Taubenberger et al. 2019).

In indentation experiments when a large planar probe is used, such as in this study, and whole sample analysis is required, major difficulty is related to the shape of bio-samples that is often not well defined and may also be non-uniform among different objects. To overcome these challenges, we suggest here a coarse grained mathematical scheme, the integrated elasticity (IE) regression, for a straightforward analysis of force measurements in varying 3D samples of indeterminate shape.

The IE regression is based on physical theories from the field of contact mechanics, which describe the elastic response of samples to exerted force in systems of well-defined geometries. When using inert surfaces, friction and adhesive interactions are often negligible. Then, different contact mechanics theories describe the force—displacement relation in good approximation as power laws (Eq. 1), where the pre-exponential factor and the exponent depend on the system geometry. The IE model assumes that samples of amorphous contour that is macroscopically concave can also be modeled using a power law, where the exponent captures morphological features of the sample and is achieved for each object from a power-law fit. This scheme is thus optimal for the mechanical analysis of tumor spheroids or other multi-cellular clusters which comprise varying geometries that usually obey those coarse assumptions. Then, the elastic moduli can be easily obtained from the expression of the pre-factor given in Eq. 4. The only geometrical pre-knowledge required for this simple analysis would be the height and length of the sample at rest.

A major advantage of the IE regression is in its extreme ease of use for analyzing force spectroscopy data. A trivial power-law fit needs to be done and the elastic moduli are then obtained by Eq. 4. Other numerical or analytical methods which describe 3D elasticity in non-trivial geometries are often not available for free. Developing such methods requires unique expertise. Moreover, other schemes are usually suitable only for systems in which the sample shape can be clearly defined (Hajji 1978; Vahabikashi et al. 2019; Karcher et al. 2003).

In the present work we studied the quality of analysis methods in mechanical measurements of tumor spheroids constructed from different types of human melanoma cells lines: either A375 or WM-255. Tumor spheroids are 3D multicellular bodies used extensively in cancer research, providing an ex vivo model of physiological tumors (Shoval et al. 2017; Nath and Devi 2016). The stiffness of the spheroids changes with the deposition of extracellular components that mimic tissue-like maturation (McKenzie et al. 2018*).* Therefore tumor spheroids mechanics may indicate on the dynamical state of the culture in respect to spatial cellular effects.

We found here that the spheroid elastic moduli predicted by linear regressions were more than twofold smaller than those obtained with the IE model, for either A375 or WM-266-4 spheroids. This is in accordance with previous studies which demonstrated that linear regressions resulted in moduli that were smaller than those obtained by well-established analysis (Baraniak et al. 2012; Conrad et al. 2019; Tietze et al. 2019). One example is a study which measured the elastic moduli of ovarian cancer nodules using either AFM or Microsquisher (a previous version of the MicroTester) (Conrad et al. 2019). The AFM measurements were done with a micro-sphere indenter and accordingly the data was fitted to the Hertz model, being a well-recognized analysis for such experiments. In the Microsquisher experiments parallel compression was done. In this case, as widely discussed here, there is lack in simple analytical methods, and the force–displacement data was fitted to a linear curve. The elastic moduli obtained using the AFM were more than seven fold higher than the moduli derived by the Microsquisher experiments. These gaps can result from the differences in the type of the measurements, as the AFM measures local elasticities, while in parallel compression global scale elasticity of the whole sample is involved. We would like to stress here that an additional reason to the different assessments can be that the linear assumption is inappropriate for the parallel compression experiments, due to the amorphous shape of the bio-samples, leading to calculated moduli that are smaller than real.

Previous studies in Mesenchymal Stem Cell (MSC) spheroids also suggest that linear regression can lead to underestimation of the elastic moduli. Microsquisher experiments that were followed by linear analysis predicted Young’s moduli in the range of 100 Pa (Baraniak et al. 2012). On the other hand, AFM indentation in MSC spheroids with a micro-bead predicted moduli that were order of magnitude higher, using Hertz model (Tietze et al. 2019). Also in this example, while the gaps in the moduli can result from deviations in the type of the measurements, they can also indicate an artifact due to inaccuracy of the linear assumption. The default usage of linear regression, even when the sample shape is not well defined, reflects the unmet need in simple regression schemes that would be more accurate for the analysis of parallel compression data. The IE model, which predicted higher moduli than the linear fitting—similar to well established analysis—provides a user friendly regression scheme that can fulfil the need to account for indeterminate geometries.

Using both analyses we found here that the A375 spheroids were one order of magnitude more elastic than the WM-266-4 spheroids. However, single cell data using AFM showed comparable elasticities of A375 cells and WM-266-4 cells (Sobiepanek et al. 2022; Bobrowska et al. 2019). This reflects the abundancy of factors which affect the mechanical properties of 3D cellular aggregates, in a manner that can be very specific to the cell type. Some examples include the spheroid density, the presence of ECM components, the 3D organization of cells, and the cell–cell or cell-ECM contacts. Moreover, both the contact area and the indentation are orders of magnitude larger in the spheroid compression measurements as compared to single cell AFM experiments. In the MicroTester experiments done here the indenting plate is much larger than spheroid size, and thus they provide collective mechanical knowledge which integrates contributions from all spheroid components (primarily cells and ECM) in a hundreds of microns’ scale. On the other hand, cellular AFM experiments can indicate on local elasticities of the membrane, the cytoskeleton cortex, or some other specific cellular compartments that are situated in the pressing zone, and within the indentation depth. In other words, the mechanical information gained by AFM experiments with cells is very local, in the sub-micron scale. Therefore, these two types of measurements are not expected to provide similar results.

Using image analysis, we quantified here two geometrical aspects of tumor spheroid deviations from the linear case: one is the macroscopic non-planarity of the sample, and the other is the surface roughness. The generality of the IE model can potentially capture both effects, however since the model is based on contact mechanics theories which assume smooth contacts, surface roughness may require further investigation (Hyun and Robbins 2007; Persson 2006; Carbone and Bottiglione 2011). Indeed we found that in WM-266-4 spheroids, which have relatively smooth surfaces, the non-planarity did not affect the fit accuracy using the IE model.

Fitting the data with the IE model resulted in higher precision in comparison with the linear fit. In spite of the deviations from the linear assumption, R^2^ of the linear fits was fine as well; however the obtained elastic moduli varied between the two models. This signified the need to carefully examine analysis methods, even when they provide valuable fitting precision. An additional critical point is that in order to validate the parameters obtained from experiments, one needs a reliable reference. However, in the case of spheroid parallel pressing, since there is no well accepted analytical analysis, there is lack in validation reference. Thus, for reliable comparison between samples, either within the same experiments or in a universal manner, we suggest that relative moduli values would be of highest validity. Universal relative values can be obtained using a well-defined reproducible reference, such as the polyacrylamide gels used here.

To summarize, while linear fit is often used in analyzing compression measurements of multi-cellular clusters (Conrad et al. 2019; Omelyanenko et al. 2020; Pradhan et al. 2017), this method may not provide satisfying reliability. Alternatively, the IE scheme is suitable as a regression method for analyzing mechanical data in multi-cellular clusters or other samples that are coarsely concave. Importantly, fitting force–displacement data with the IE model is extremely simple and fast, and is free, unlike other existing theories. The reliability of quantitative comparison between different types of spheroids can further be improved by regarding relative (rather than absolute) moduli, using universal reference samples.

## Abbreviations

3D: Three dimensional
IE: Integrated elasticity
AFM: Atomic force microscopy
